# The tetraspanin transmembrane protein CD53 mediates dyslipidemia and integrates inflammatory and metabolic signaling in hepatocytes

**DOI:** 10.1016/j.jbc.2022.102835

**Published:** 2022-12-27

**Authors:** Cassandra B. Higgins, Joshua A. Adams, Matthew H. Ward, Zev J. Greenberg, Małgorzata Milewska, Jiameng Sun, Yiming Zhang, Luana Chiquetto Paracatu, Qian Dong, Samuel Ballentine, Weikai Li, Ilona Wandzik, Laura G. Schuettpelz, Brian J. DeBosch

**Affiliations:** 1 Department of Pediatrics, Washington University School of Medicine, St Louis, Missouri, USA; 2 Department of Chemistry, Washington University in St Louis, St Louis, Missouri, USA; 3 Biotechnology Center, Silesian University of Technology, Gliwice, Poland; 4 Department of Organic Chemistry, Bioorganic Chemistry and Biotechnology, Faculty of Chemistry, Silesian University of Technology, Gliwice, Poland; 5 Department of Pathology and Immunology, Washington University School of Medicine, St Louis, Missouri, USA; 6 Department of Biochemistry & Molecular Biophysics, Washington University School of Medicine, St Louis, Missouri, USA; 7 Siteman Cancer Center, Washington University, St. Louis, Missouri, USA; 8 Department of Cell Biology & Physiology, Washington University School of Medicine, St Louis, Missouri, USA

**Keywords:** tetraspanin, CD53, fasting, caloric restriction, energy metabolism, insulin resistance, glucose transport, GLUT, nonalcoholic fatty liver disease, proteomics, thermogenesis, obesity, FGF21, trehalose, lactotrehalose, AIBN, 2,2′-azobis(2-methylpropionitrile), AMPK, AMP-activated protein kinase, BSA, bovine serum albumin, BW, body weight, cDNA, complementary DNA, CTA, chain transfer agent, DMSO, dimethyl sulfoxide, EGFR, epidermal growth factor receptor, eWAT, epididymal white adipose tissue, FA, fatty acid, FGF21, fibroblast growth factor 21, [3H]-2DG, [3H]-2-deoxy-d-glucose, GLUT8, glucose transporter 8, GO, Gene Ontology, HEA, 2-hydroxyethyl acrylate, IL, interleukin, LPS, lipopolysaccharide, NAFLD, nonalcoholic fatty liver disease, NASH, nonalcoholic steatohepatitis, NIH, National Institutes of Health, PGC1α, peroxisome proliferator antigen receptor gamma coactivator-1-alpha, TG, triglyceride, TNFα, tumor necrosis factor alpha, TreA, 6-O-acryloyl-trehalose, WD, Western diet

## Abstract

Tetraspanins are transmembrane signaling and proinflammatory proteins. Prior work demonstrates that the tetraspanin, CD53/TSPAN25/MOX44, mediates B-cell development and lymphocyte migration to lymph nodes and is implicated in various inflammatory diseases. However, CD53 is also expressed in highly metabolic tissues, including adipose and liver; yet its function outside the lymphoid compartment is not defined. Here, we show that CD53 demarcates the nutritional and inflammatory status of hepatocytes. High-fat exposure and inflammatory stimuli induced CD53 *in vivo* in liver and isolated primary hepatocytes. In contrast, restricting hepatocyte glucose flux through hepatocyte glucose transporter 8 deletion or through trehalose treatment blocked CD53 induction in fat- and fructose-exposed contexts. Furthermore, germline CD53 deletion *in vivo* blocked Western diet–induced dyslipidemia and hepatic inflammatory transcriptomic activation. Surprisingly, metabolic protection in CD53 KO mice was more pronounced in the presence of an inciting inflammatory event. CD53 deletion attenuated tumor necrosis factor alpha–induced and fatty acid + lipopolysaccharide-induced cytokine gene expression and hepatocyte triglyceride accumulation in isolated murine hepatocytes. *In vivo*, CD53 deletion in nonalcoholic steatohepatitis diet-fed mice blocked peripheral adipose accumulation and adipose inflammation, insulin tolerance, and liver lipid accumulation. We then defined a stabilized and trehalase-resistant trehalose polymer that blocks hepatocyte CD53 expression in basal and over-fed contexts. The data suggest that CD53 integrates inflammatory and metabolic signals in response to hepatocyte nutritional status and that CD53 blockade may provide a means by which to attenuate pathophysiology in diseases that integrate overnutrition and inflammation, such as nonalcoholic steatohepatitis and type 2 diabetes.

The tetraspanins represent an expansive and diverse family of membrane-spanning receptors that mediate multiple processes across several cell types. These proteins have both intracellular and extracellular domains and act in concert as part of microdomains with other membrane surface proteins to regulate adhesion, migration, cellular fusion, proliferation, and signaling (1, 2, 3, 4, 5). Among this array of functions, some tetraspanins can mediate development and function, including inflammatory processes in lymphocytes (1, 2).

One member, CD53/MOX44/TSPAN25, is a tetraspanin that has incompletely characterized function (6). Prior data indicate that CD53 mediates B-cell development and for recirculation and homing of both B and T cells to lymph nodes (7, 8). Similarly, CD53 deficiency impaired neutrophil transmigration, thus CD53 likely plays an important role in the adaptive immune response (9). However, CD53 is expressed across the lymphoid compartment and is thus likely to serve a broader immune function than the current literature suggests. For example, from a disease-specific point of view, CD53 is upregulated in atherosclerotic plaques and during coronary revascularization (10, 11, 12) and is implicated in cancer surveillance and regulation of innate circulating tumor necrosis factor alpha (TNFα) levels in humans (13). Accordingly, CD53 deficiency in humans predisposes to multiple recurrent infections (14), suggesting broad immune participation for CD53. In addition, whereas CD53 is most highly expressed in the immune compartment, it is also expressed outside the immune compartment, including in highly metabolic peripheral tissues, such as liver and adipose tissues (15). Moreover, the function of CD53 outside the immune compartment is not well understood (15). Thus, potential CD53 functions in metabolic tissue remain unaddressed.

Nonalcoholic fatty liver disease (NAFLD) is a disease of overnutrition in the liver, characterized by dysregulated fat accumulation. This is manifest on a spectrum that ranges from simple steatosis to frank lobular inflammation and hepatocyte ballooning, known as nonalcoholic steatohepatitis (NASH) (16). It is the most common chronic liver disease worldwide and is becoming the most common cause for liver transplantation in the United States (17, 18, 19). Beyond the liver, NAFLD and NASH are associated with development of type 2 diabetes mellitus, cardiovascular disease, and all-cause mortality, making this both a common and morbid disease.

Consistent with the fact that NAFLD stems in part from overnutrition, we and others have shown that mimicking the hepatocyte fasting–like response through glucose transport blockade induces compensatory adaptive processes that can be leveraged in contexts of overnutrition. These adaptations include activation of nitrogen catabolism and autophagic flux, activation of the AMP-activated protein kinase (AMPK) pathway, secretion of the antidiabetic, insulin-sensitizing hepatokine, fibroblast growth factor 21 (FGF21 (20, 21, 22, 23, 24, 25, 26)), NAD^+^ salvage (27, 28, 29, 30, 31), and activation of key fasting-state transcriptional regulators—transcription factor EB and peroxisome proliferator antigen receptor gamma coactivator-1-alpha (PGC1α) (16, 31, 32, 33, 34, 35, 36, 37, 38, 39, 40, 41, 42, 43, 44, 45, 46, 47, 48). Novel glucose transporter inhibitors can drive this program to attenuate NAFLD, including trehalose, lactotrehalose, and other glucosides (35, 36, 37, 39, 40, 49, 50, 51, 52, 53, 54, 55, 56).

NASH represents the inflammatory end of the NAFLD spectrum, specifically characterized by lobular inflammation and hepatocyte ballooning in response to overnutrition, lipid overload, circulating cytokines, and other factors (16, 57, 58, 59). B- and T cell-mediated adaptive immunity has a newly recognized role in driving NASH pathophysiology. Specifically, both human and rodent NASH models feature B- and T-cell infiltration in the liver, and blocking lymphocyte activation and recruitment ameliorated steatohepatitis and fibrosis in mouse models (60). Accordingly, recent transcriptomic studies identified CD53 as a potential immune biomarker for NASH (61). CD53 expression in adipose tissue correlated to levels of hepatic steatosis, as quantified by hepatic triglyceride (TG) levels in a panel of mouse strains, and is associated with adipose tissue inflammation in previous studies of obesity (61, 62). Together, these data prompted the hypothesis that CD53 moderates both inflammatory and metabolic functions under duress to promote inflammatory metabolic diseases, such as insulin resistance, visceral adipose inflammation, and hepatic lipid accumulation.

Here, we show that both inflammatory and nutritional stimuli induce hepatocyte CD53. We interdict this by inhibiting carbohydrate substrate uptake by hepatocytes by treating them with the glucose uptake inhibitor trehalose or by deleting the hepatocyte carbohydrate carrier, glucose transporter 8 (GLUT8) (34, 35, 38). We further show that CD53 cell-autonomously mediates TNFα and lipopolysaccharide (LPS) proinflammatory signaling in hepatocytes. *In vivo*, targeted germline CD53 deletion protected against Western diet (WD)–induced hepatic inflammatory gene expression, and NASH diet–induced peripheral fat, hepatic lipid accumulation, and insulin intolerance. We then identify a trehalose polymer, pTreA40, which induces hepatocyte fasting–like signaling *via* AMPK, FGF21, PGC1α, and Arg2 signaling (31, 35, 39, 44, 46, 47, 51) and blocks CD53 expression in hepatocytes. We conclude that CD53 function assumes metabolic and inflammatory functions in hepatocytes under metabolic and proinflammatory duress.

## Results

### Overnutrition and inflammatory stimuli induce hepatocyte CD53 *in vitro* and *in vivo*

**Figure 1:**
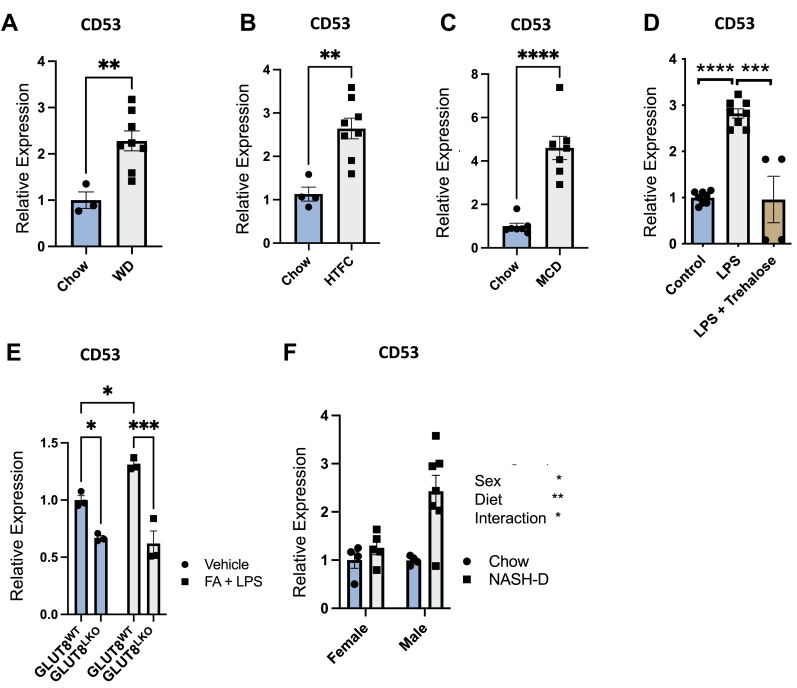
**Dietary and inflammatory stress induce CD53 in murine liver and isolated hepatocytes.***A*–*C*, quantitative RT–PCR quantification of liver CD53 expression in chow-fed mice *versus* mice fed (*A*) Western diet (WD), (*B*) high transfat and cholesterol (HTFC) diet, or (*C*) methionine- and choline-deficient diet (MCD). *D*, CD53 expression in isolated primary murine hepatocytes treated with lipopolysaccharide (LPS) in the presence or the absence of 100 mM trehalose. *E*, CD53 expression in isolated primary murine hepatocytes from WT and liver-specific GLUT8-deficient mice (GLUT8^LKO^) treated with vehicle or with albumin-conjugated nonesterified fatty acids and LPS. *F*, liver CD53 expression in WT male and female mice fed with either chow diet or WD with low-dose, weekly CCL_4_ (NASH-D) to induce NASH. ∗*p* < 0.05, ∗∗*p* < 0.01, ∗∗∗*p* < 0.001, and ∗∗∗∗*p* < 0.0001 by t-tailed *t* test (*A*–*C*), one-way ANOVA with Dunnett’s multiple comparisons test (*D*), and by two-way ANOVA (*E* and *F*). A genotype main effect was detected by two-way ANOVA in (*E*). Sex, diet, and their interactions were significant by two-way ANOVA in (*F*). GLUT8, glucose transporter 8; NASH-D, nonalcoholic steatohepatitis diet.

We first tested the effect of obesigenic and inflammatory diet (63) on hepatic CD53 expression. About 16 weeks of WD feeding (Fig. 1*A*), 12-week high-transfat feeding (Fig. 1*B*), and 4-week methionine and choline-deficient diet feeding (Fig. 1*C*), each induced hepatic CD53 expression twofold to fourfold in mice. CD53 induction in the setting of proinflammatory overnutrition prompted us to test in isolated murine hepatocyte cultures if glucose transporter blockade and induced fasting-like responses also blocked CD53 expression. We treated isolated primary murine hepatocytes with LPS and with or without trehalose (24 h, 100 mM). LPS upregulated CD53 expression, and this was abrogated with concomitant trehalose treatment (Fig. 1*D*). One mechanistic target of trehalose is GLUT8 (34, 40), and so we examined if hepatocyte-specific GLUT8 deletion also blocked CD53 induction in our *in vitro* inflammatory overnutrition model (bovine serum albumin [BSA]–conjugated fatty acids + LPS [FA+ LPS]). FA + LPS induced CD53, and both basal as well as stimulated GLUT8LKO hepatocytes exhibited significantly lower CD53 expression (*p* < 0.05 for genotype-stimulus interaction by two-way ANOVA, Fig. 1*E*). We then tested if CD53 regulation is sexually dimorphic by feeding chow or WD with low-dosage carbon tetrachloride (hereafter, “NASH Diet,” NASH-D) for both male and female mice (12 weeks, Fig. 1*F*, (64)). We detected both sex and diet main effects as well as a significant diet–sex interaction dictating CD53 expression. Overall, the data establish that CD53 expression is dimorphic, and induced in liver following nutritional overload and inflammatory stimuli. Its expression correlates with exacerbated and attenuated liver pathology.

### CD53 mediates WD-induced dyslipidemia and inflammatory transcriptional activation in liver

**Figure 2:**
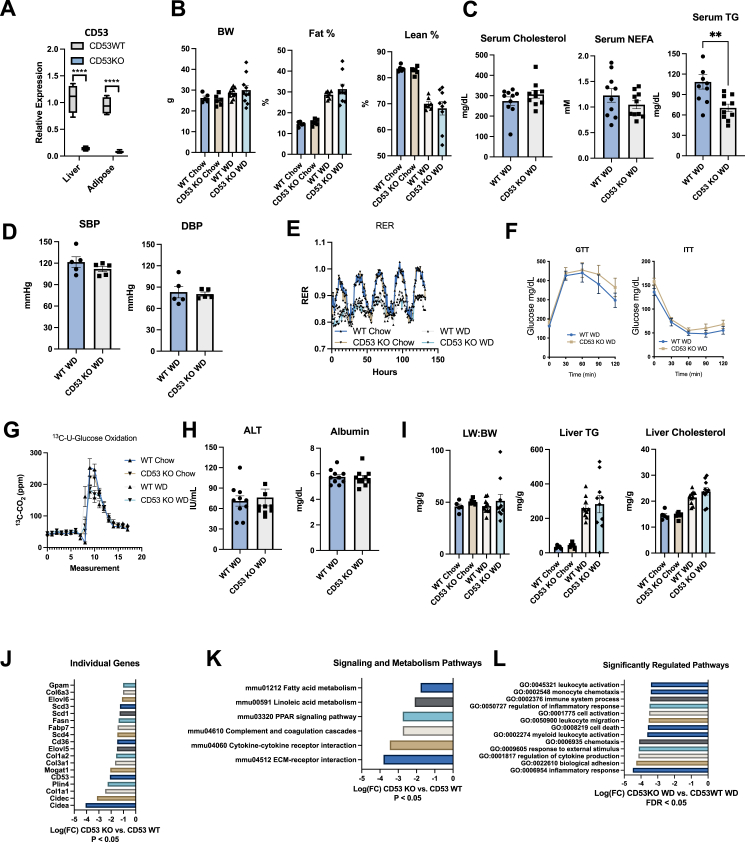
**CD53 mediates Western diet (WD)–induced hypertriglyceridemia and inflammatory transcriptional activation in liver.***A*, quantitative RT–PCR quantification of CD53 transcript in liver and epididymal white adipose tissue in n = 3 to 5 chow-fed mice per group. *Boxes* represent 75%ile and 25%ile values. *Middle bar* represents median values. *Whiskers* represent range. ∗∗∗∗*p* < 0.0001 by two-tailed *t* test *versus* WT sample. *B*, body weight (BW) and body composition in WT and CD53 KO mice fed 12 weeks WD. *C*, circulating lipids in nonfasting chow- and WD-fed WT and CD53 KO mice. ∗∗*p* < 0.01 by two-tailed *t* test *versus* WT sample. *D*, systolic blood pressure (SBP) and diastolic blood pressures (DBP) in nonfasting chow- and WD-fed WT and CD53 KO mice. *E*, indirect calorimetric quantification of respiratory exchange ratio (RER) in chow- and WD-fed WT and CD53 KO mice during light and dark cycle. *F* and *G*, glucose tolerance testing (GTT) and insulin tolerance testing (ITT) and glucose oxidation of intraperitoneal and universally labeled ^13^C-glucose in chow- and WD-fed WT and CD53 KO mice. *H* and *I*, serum transaminases, albumin, liver weight-to-body weight ratio, and colorimetric quantification of liver lipids in chow- and WD-fed WT and CD53 KO mice. *J*–*L*, transcriptomic analysis of significantly regulated genes, signaling/metabolism pathways (*J* and *K*, *p* < 0.05) and overall significantly regulated gene pathways (*L*, false discvovery rate [FDR] <0.05) in livers from n = 2 to 4 WD-fed CD53 KO mice fed relative to WD-fed WT mice, threshold *p* < 0.05 (*J* and *K*) and FDR <0.05 (in *L*). ∗*p* < 0.05, ∗∗*p* < 0.01, ∗∗∗*p* < 0001, ∗∗∗∗*p* < 0.0001 *versus* bracketed control group by two-tailed *t* test (in *A* and *C*).

Because CD53 regulation correlates with metabolic pathology and treatment of that pathology in liver and in isolated hepatocytes, we examined CD53 expression in chow-fed WT and germline CD53-deficient (CD53 KO) mice (6, 7, 8). We confirmed loss of CD53 in whole liver and whole epididymal white adipose tissue (eWAT) relative to CD53 WT mice (Fig. 2*A*). These particular tissues were assayed because of their high metabolic capacity and because prior data demonstrate CD53 upregulation in the obese state (Fig. 2*A*, (61, 62)). We used these CD53-deficient mice to define if CD53 mediates hepatic responses to chronic overnutrition. To that end, we placed male WT and CD53 KO mice on chow or 12-week WD (42% kcal from fat). Chow and WD-fed WT and CD53 KO mice exhibited similar endpoint body weight (BW) and body composition (Fig. 2*B*), fasting serum cholesterol and nonesterified FAs (Fig. 2*C*), blood pressure, and respiratory exchange ratio when placed in a metabolic chamber (Fig. 2, *D* and *E*). However, fasting serum TGs were significantly reduced in WD-fed CD53 KO mice (Fig. 2*C*). Quantification of glucose homeostasis revealed similar glucose and insulin tolerance testing curves (Fig. 2*F*) as well as glucose ^13^C-glucose oxidation curves (Fig. 2*G*). Liver assessments revealed similar serum alanine aminotransferase and albumin (Fig. 2*H*) and liver weight-to-BW ratio, liver TG, and liver cholesterol (Fig. 2*I*). Yet, detailed transcriptomic analysis revealed decreased expression of genes involved in lipid synthesis (Plin2, FASN, GPAM SCD3, Elovl5, Elovl6, MOGAT1, and SCD1) and in inflammation and fibrosis (*e.g.*, Col1A1, Col1A2, Col3A1, and Col6A3) in CD53 KO mouse liver (Fig. 2*J*). Alignment of these genes into signaling and metabolism-specific pathways by Gene Ontology (GO) analysis revealed significantly downregulated signaling- and metabolism-specific pathways in WD-fed CD53 KO liver, which included FA metabolism, linoleate metabolism, complement cascades, cytokine–cytokine receptor interaction, and extracellular matrix–receptor interaction (Fig. 2*K*). This was paralleled by analysis of pathways among the most downregulated overall, which included leukocyte activation, monocyte chemotaxis, regulation of inflammation, cell death, myeloid activation, cytokine production, and inflammatory response (Fig. 2*L*).

### CD53 mediates TNFα-induced inflammatory gene induction

**Figure 3:**
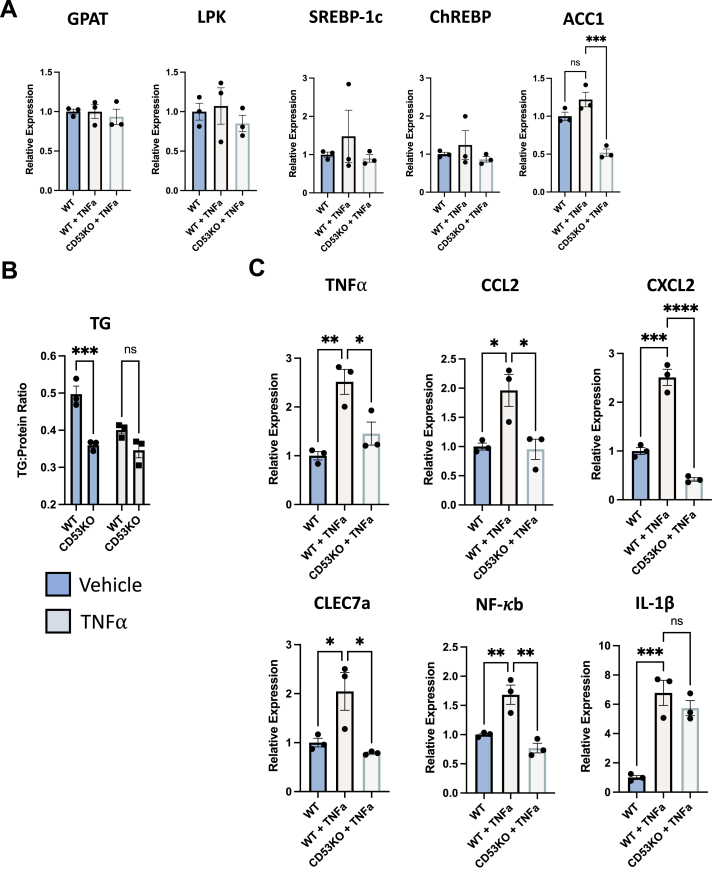
**CD53 mediates hepatocyte tumor necrosis factor alpha (TNFα) signaling.***A*, expression of *de novo* lipogenic genes, glycerol phosphate acyltransferase (GPAT), liver pyruvate kinase (LPK), sterol response element binding protein-1c (SREBP-1c, carbohydrate response element–binding protein (ChREBP), and acetyl coA carboxylase 1 (ACC1) in isolated primary hepatocytes from WT and CD53 KO mice treated with or without recombinant mouse TNFα. *B* and *C*, triglycride (TG) accumulation (*B*) and inflammatory marker gene expression (*C*) in isolated hepatocytes from WT and CD53 KO mice treated with or without TNFα. ∗, ∗∗, ∗∗∗, and ∗∗∗∗ *p* < 0.05, *p* < 0.01, *p* < 0001, *p* < 0.0001 *versus* bracketed control group by one-way ANOVA with Dunnett’s multiple comparison testing (*A* and *C*) or by two-way ANOVA (in *B*) with Sidak’s multiple comparison testing.

Reduced molecular signatures of liver inflammation *in vivo* prompted evaluation of the hepatocyte-specific response to inflammatory stimuli. We therefore subjected isolated primary murine hepatocytes from WT and CD53 KO mice to vehicle or recombinant murine TNFα. As anticipated, TNFα had minimal effect *per se* on *de novo* lipogenic gene expression (GPAT, LPK, ACC1, SREBP-1C, and ChREBP) and *in vitro* TG accumulation (Fig. 3, *A* and *B*), although CD53 KO hepatocytes exhibited lower ACC1 expression after TNFα treatment and lower *in vitro* TG accumulation both at baseline and under TNFα stimulation (genotype main effect *p* = 0.0003 for WT *versus* CD53 KO hepatocytes by two-way ANOVA, Fig. 3*B*). In contrast, TNFα treatment robustly increased inflammatory hepatocyte gene expression in WT hepatocytes, including TNFα, CCL2, CXCL2, Clec7A, and NF-κb (Fig. 3*C*). This inflammatory gene upregulation was largely disrupted in CD53 KO hepatocytes. The gene induction response to interleukin 1β (IL-1β) was preserved, suggesting that the TNFα signaling blockade because of CD53 deletion retains some signal specificity (Fig. 3*C*).

### CD53 mediates FA-induced TG accumulation and inflammatory gene expression in an *in vitro* NASH model

**Figure 4:**
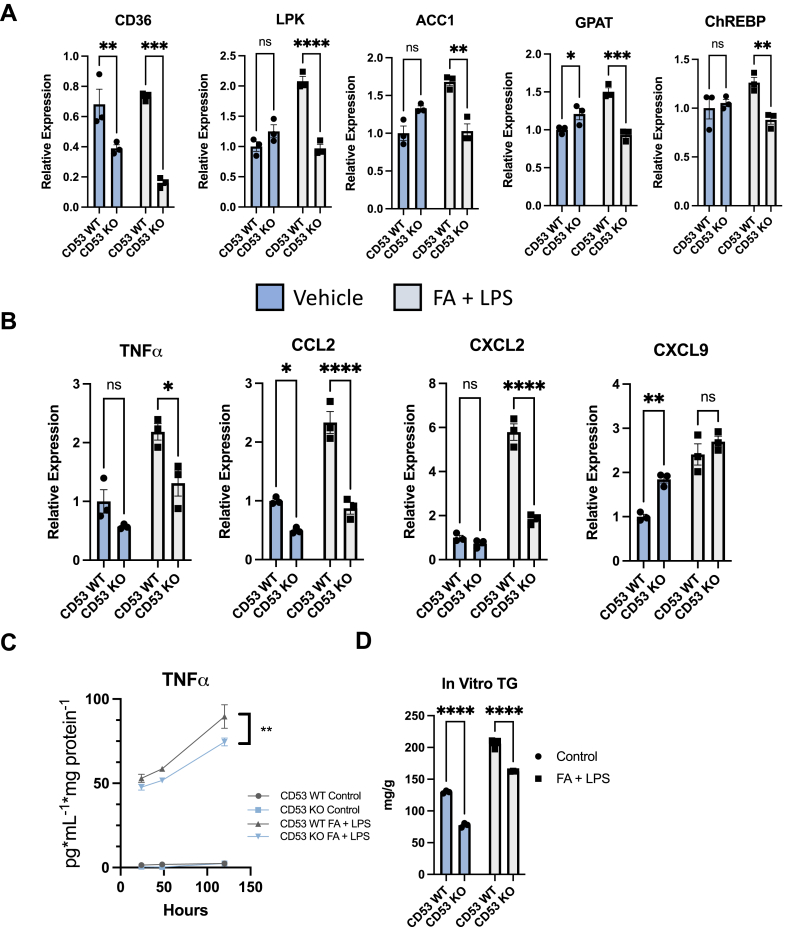
**Protection from inflammatory gene induction and triglycride (TG) accumulation in an *in vitro* nonalcoholic steatohepatitis (NASH) model.***A* and *B*, *de novo* lipogenic and inflammatory gene expression in primary hepatocytes from WT and CD53 KO mice treated with or without bovine serum albumin (BSA)-conjugated fatty acids (FAs) + lipopolysaccharide (LPS). *C*, enzyme-linked immunosorbent assay data showing lower tumor necrosis factor alpha (TNFα) secretion into the extracellular media after treatment with FA + LPS for 24, 48, and 120 h. ∗∗*p* < 0.01 for genotype main effect in CD53 KO FA + LPS *versus* WT FA + LPS groups. *D*, quantitative triglycerides in n = 3 primary hepatocytes from WT and CD53 KO mice treated with or without BSA-conjugated fatty acids and LPS. ∗, ∗∗, ∗∗∗, and ∗∗∗∗*p* < 0.05, 0.01, 0.001, 0.0001 *versus* bracketed control by two-way ANOVA with Sidak’s multiple comparison testing in *A*, *B*, and *D*.

The consistent anti-inflammatory protection in liver in an overnutrition model in CD53 KO mice *in vivo* (Fig. 2) and the robust protection against TNFα-induced cytokine gene expression in CD53 KO isolated hepatocytes (Fig. 3) led us to examine how CD53-deficient hepatocytes respond to combined metabolic and inflammatory duress, as is seen in NASH. Therefore, we utilized our established *in vitro* NASH model, wherein we treat primary hepatocytes from CD53 WT and CD53 KO mice with BSA alone or BSA-conjugated FAs + LPS. FA + LPS induced *de novo* lipogenic genes LPK, ACC1, GPAT, and ChREBP (Fig. 4*A*). CD53-deficient hepatocytes failed to upregulate these DNL-related genes in response to FA + LPS (Fig. 4*A*). SREBP-1c was not induced under these conditions, and neither SREBP-1c nor SCD1 expression was lower in CD53 KO hepatocytes when compared with CD53 WT cultures (Fig. S1). In addition, a related membrane-bound protein, CD36 (65), was decreased in both BSA-treated and FA + LPS-treated hepatocytes (Fig. 4*A*). Moreover, CD53 KO hepatocytes were again protected from FA + LPS-induced CCL2, CXCL2, and TNFα expression relative to FA + LPS-treated CD53 WT hepatocytes (Fig. 4*B*). CXCL9 expression was not lower in WT and CD53 KO hepatocytes. This indicated a relatively selective defect in FA + LPS inflammatory signaling in the absence of CD53 in hepatocytes (Fig. 4*B*). We corroborated inflammatory gene expression data by measuring secreted TNFα protein by ELISA in the culture media of isolated hepatocytes treated with or without FA + LPS (24, 48, and 120 h treatment). Throughout time course of treatment, FA + LPS-treated CD53 KO hepatocytes accumulated lower TNFα in the extracellular medium when compared with CD53 WT controls (Fig. 4*C*). Finally, CD53 KO hepatocytes accumulated significantly lower TG in response to FA + LPS (Fig. 4*D*). These data overall indicate that CD53 mediates stimuli that integrate overnutrition and inflammation.

### CD53 deficiency protects against overnutrition and inflammatory stimuli in eWAT

**Figure 5:**
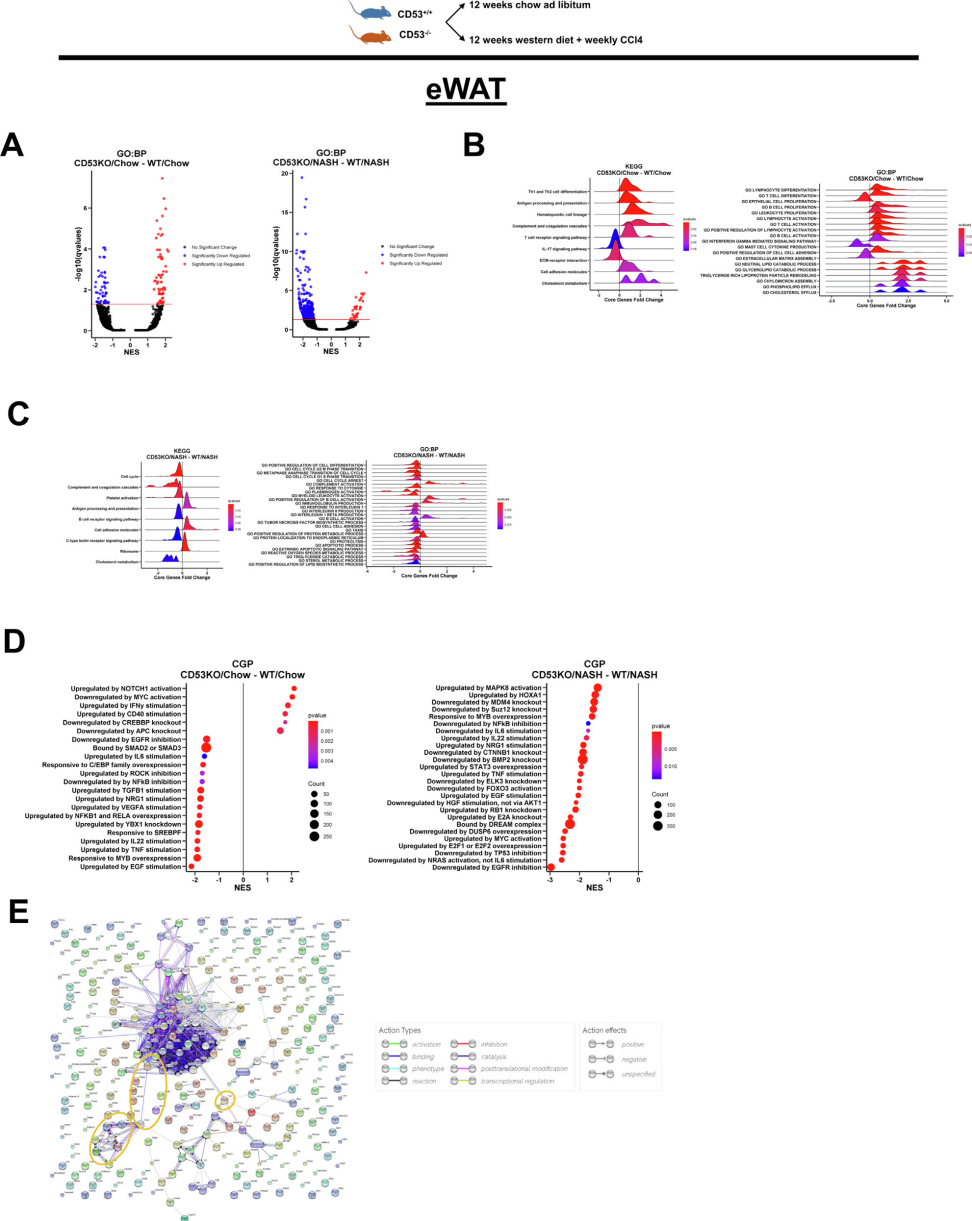
**CD53 mediates peripheral adipose inflammation during diet-induced metabolic and inflammatory duress.***A*, volcano plot showing differentially regulated genes in epididymal white adipose tissue (eWAT) from mice following 12 weeks exposure to either chow- or NASH-D. *B*, gene set enrichment analyses (GSEAs) showing differentially regulated Kyoto Encyclopedia of Genes and Genomes (KEGG) and Gene Ontology (GO): biological process pathways in eWAT from chow-fed CD53 KO *versus* WT mice. *C*, GSEAs showing perturbed KEGG and GO: biological process pathways in eWAT from NASH D-fed CD53 KO *versus* WT mice. *D*, GSEA implicating signaling and transcription factor modulation, using the MSigDB Chemical and Genetic Perturbations (CGP) database and eWAT transcriptomics datasets introduced above, with selected results pulled out as ridge plots. *E*, STRING plot visualizing interactions between all significantly altered transcripts in eWAT from CD53 KO *versus* WT mice fed NASH-D. Tumor necrosis factor alpha (TNFα) pathway-relevant interactions are circled in *yellow*. NASH-D, nonalcoholic steatohepatitis diet.

To interrogate the CD53 KO response to metabolic inflammation *in vivo*, we treated WT and CD53 KO mice to NASH-D (12 weeks) as an established model of NASH (64). To first assess the function of CD53 in adipose inflammation, we subjected eWAT from NASH diet–fed WT and CD53 KO mice to bulk transcriptomic analysis. Volcano plot analysis of bulk eWAT transcriptomics revealed minimal basal differences from chow-fed mice when comparing WT and CD53 KO mice, but several more significantly altered genes were downregulated in NASH-fed CD53 KO eWAT *versus* WT eWAT (Fig. 5*A*). Basal differences included downregulation of IL-17 signaling and extracellular matrix–receptor interaction and upregulation of cholesterol metabolism (Fig. S2). Surprisingly, antigen processing, TH_1_/TH_2_ differentiation, and T-cell receptor signaling were induced at baseline in CD53 KO eWAT (Fig. 5*B*). Yet, under NASH-fed conditions, CD53 KO eWAT exhibited reduced c-type lectin receptor signaling, B-cell receptor signaling, platelet activation, and complement/coagulation cascades, and upregulated cholesterol metabolism pathways (Figs. 5*C* and S2). MSigDB-mediated predictive mapping of signaling perturbations that unify these pathways, and which remained consistently downregulated under both chow and NASH diet–fed conditions, included downregulated epidermal growth factor/epidermal growth factor receptor (EGFR), TNFα, IL-22, and NF-κB signaling (Fig. 5*D*). We corroborated connectivity of TNFα and EGFR signaling in conjunction with other significantly regulated mediators in eWAT by STITCH analysis *in silico* (Fig. 5*E*). Thus, agnostic analyses implicate TNFα and EGFR signaling as potential regulatory nodes downstream of CD53 signaling to explain transcriptomic changes observed in eWAT from CD53 KO *versus* WT mice after NASH-D.

### CD53 deletion protects against diet-induced adipose tissue and liver lipid accumulation as well as insulin resistance

**Figure 6:**
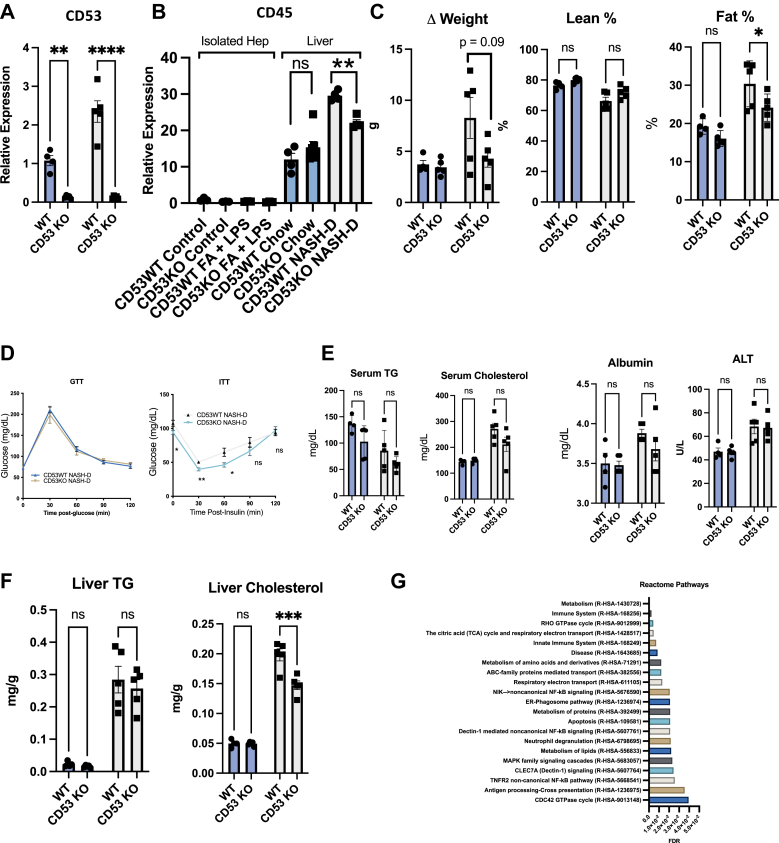
**CD53 mediates nonalcoholic steatohepatitis (NASH)-diet (NASH-D)–induced lipid and glucose metabolic derangements in peripheral and liver lipids and insulin intolerance.***A*, CD53 expression in WT and CD53 KO livers from mice fed chow or NASH-D. *B*, quantitative RT–PCR analysis of CD45 mRNA in WT and CD53 KO isolated hepatocytes treated with or without fatty acid (FA) + lipopolysaccharide (LPS) (24 h), and in liver from WT and CD53 KO mice fed chow or NASH-D. ∗∗*p* < 0.01 by two-way ANOVA with Sidak’s post hoc correction for multiple comparisons (CD53 KO NASH-D *versus* WT NASH-D). *C*, body weight, % lean, and % fat mass in WT and CD53 KO mice fed chow or NASH-D. Main effects: Fat % main effect genotype factor *p* = 0.0192, diet factor, *p* <0.0001, lean % genotype factor *p* = 0.0184, diet factor *p* < 0.0001. *D*–*F*, glucose and insulin tolerance testing, serum lipids albumin and ALT, and liver lipids in WT and CD53 KO mice fed chow or NASH diet. Main effect for serum triglyceride (TG) was significant for CD53 NASH-D, *p* = 0.0326 *versus* WT NASH-D. ∗∗∗*p* < 0.001 by two-way ANOVA with Sidak’s post hoc correction for multiple comparisons *versus* bracketed WT control. *G*, significantly enriched Reactome pathways identified by peptide coimmunoprecipitation and mass spectrometry using GFP-tagged CD53 as bait and using GFP alone as a negative control. Positive interacting partners identified were then entered into the Reactome pathway database. The most significantly enriched pathways are listed.

We next turned to hepatic and whole-body metabolic characterization of WT and CD53 KO mice. We confirmed loss of CD53 expression in NASH-D-fed liver in CD53 KO mice (Fig. 6*A*). We then assessed liver for content of CD45 by mRNA expression, as a general lymphocyte marker. CD45 expression was not detectable in our isolated murine hepatocyte cultures under basal and FA + LPS-treated conditions (Fig. 6*B*). However, in livers *in vivo*, CD45 was significantly upregulated in NASH-D-fed *versus* chow-fed mouse liver from WT mice. In contrast, liver CD45 expression was significantly blunted in NASH-D-fed CD53 KO *versus* WT mice (Fig. 6*B*). Moreover, CD53 KO mice were protected from NASH-D-induced increases in body fat content, with trends toward increased percent lean mass and lower BW gain (Fig. 6*C*). Improved body composition was paralleled by significantly improved glucose status during insulin tolerance testing, whereas glucose tolerance testing, and serum lipids, albumin, and transaminases were largely unchanged (Fig. 6, *C*–*E*). Lipid measurements revealed that liver TG was unchanged in WT *versus* CD53 KO liver, although CD53-deficient mice were protected from NASH-D-induced liver cholesterol accumulation (Fig. 6*F*) in the absence of total NAFLD activity score changes, as assessed by treatment-blinded histopathology (Fig. S3). CD53 deficiency thus improved WAT as well as liver and glucose metabolic functions in context of an overnutrition/proinflammatory diet.

We next performed immunoprecipitation and mass spectroscopic–based proteomics in isolated hepatocytes to obtain molecular insights into CD53 function. We used a GFP-tagged CD53 construct as bait in hepatocytes treated with or without conjugated FAs and LPS. As a negative control, we transfected cells with GFP alone and treated with or without FA + LPS prior to GFP-specific immunoprecipitation and mass spectroscopic analysis. We applied filtering criteria identical to those published recently (6) to define protein binding hits after immunoprecipitation. Specifically, we required identification of at least two unique peptides binding to the putative binding partner. In addition, we established that total protein abundance for each putative binding hit was at least fourfold than that observed in the negative (GFP-only) control.

First, similar to recent CD53 binding studies, we identified known CD53-binding inflammatory mediators. This included, most notably, guanine nucleotide-binding protein G(s) subunit alpha isoforms (GNAS), which mediates LPS-mediated signaling in hepatocellular carcinoma models (6). We also recapitulated prior findings by Dunlock *et al.* (6), which demonstrate that CD53 binds ARPC family members, ARPC1a and ARPC5L (Data File S1).

In addition, we newly identified CD53 binding partners in hepatocytes in FA + LPS-stimulated hepatocytes. This includes SOD2 and EHD1, which suppress inflammation and mediate macrophage CSF1R signaling, respectively. Surprisingly, we also identified multiple metabolically important factors, such as the methyltriglyceride transfer protein 1 (MTTP, a membrane-spanning lipid efflux protein) and NADH dehydrogenases, Ndufa5 and Ndufs1 (Data File S1). Agnostic categorization of all hits in the basal unstimulated state did not reveal any significantly enriched pathways as determined by protein–protein interaction (REACTOME) pathway enrichment. However, following FA + LPS treatment in hepatocytes, the most significantly enriched CD53 binding partners categorized into the metabolism and immune system pathways (Fig. 6*G*, R-HSA-1430728, R-HSA-168256). These were followed by RHO GTPase cycle (R-HSA-9012999), tricarboxylic acid cycle (R-HSA-142851), electron transport chain (R-HSA-168249), and innate immune system (R-HSA-1643685, false discovery rate <0.01 for all aforementioned pathways). Thus, by leveraging a protein interactomic-based approach, the data corroborate physiological, biochemical, transcriptional, and transcriptomic data that indicate both inflammatory and metabolic functions for hepatocyte CD53, potentially through protein–protein interaction with effector proteins in each pathway.

### Trehalose polymers induce hepatocyte fasting–like signaling and blocks CD53

**Figure 7:**
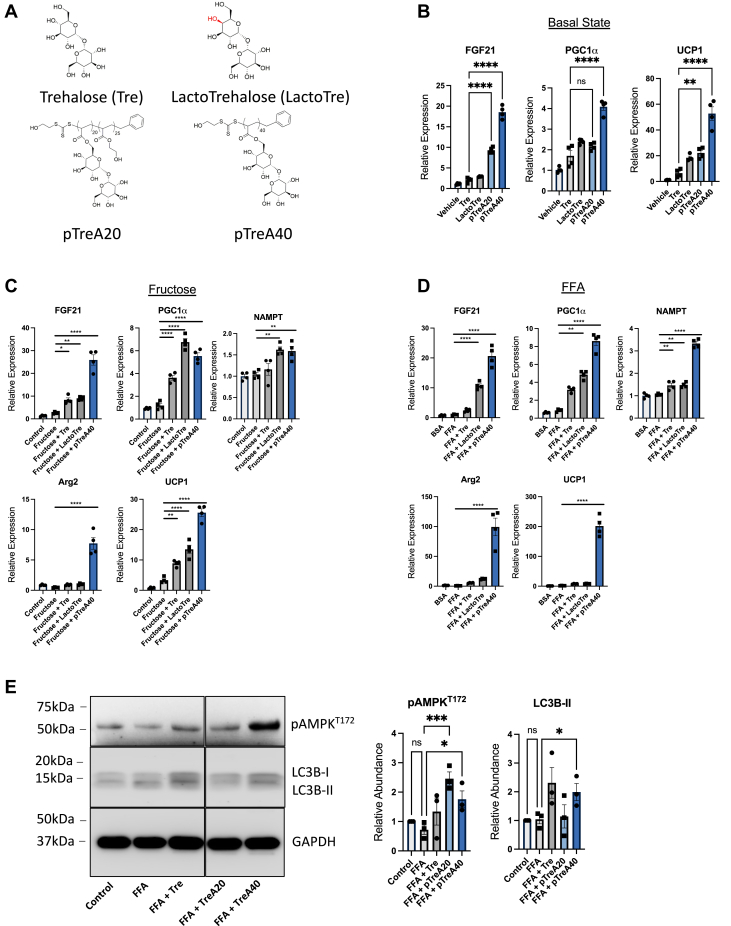

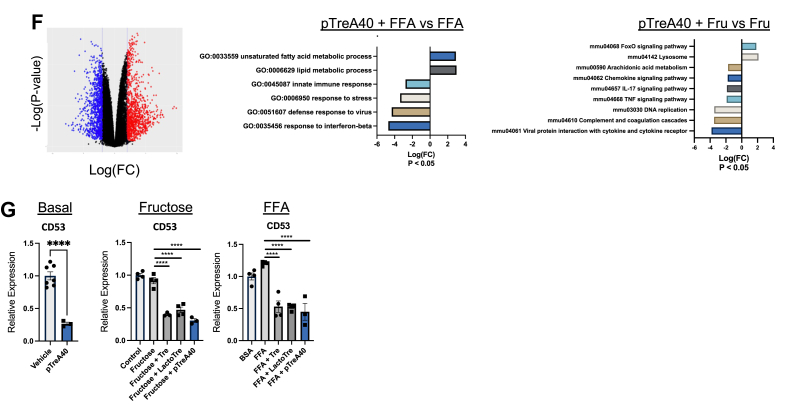
**Trehalose polymer TreA40 incites the hepatocyte fasting response and blocks CD53 expression.***A*, structure of trehalose, lactotrehalose, and polymers pTreA20 and pTreA40. *B*, quantitative RT–PCR (qRT–PCR) analysis of fasting-induced gene expression—FGF21, PGC1α, and UCP1—in isolated primary murine hepatocytes treated with trehalose, lactotrehalose, pTreA20, and pTreA40 (100 mM carbohydrate content per compound). *C* and *D*, qRT–PCR analysis of fasting-induced gene expression—FGF21, PGC1α, Arg2, NAMPT, and UCP1—in isolated primary murine hepatocytes treated with trehalose, lactotrehalose, pTreA20, and pTreA40 with or without 25 mM fructose (*C*) or bovine serum albumin (BSA)-conjugated fatty acids (FAs; *D*). *E*, immunoblot demonstrating increased AMPK (T172) phosphorylation, and LC3B-II accumulation in primary hepatocytes treated with or without BSA-conjugated FA in the presence or the absence of trehalose, pTreA20 or pTreA40. *F*, volcano plot of RNA-Seq data demonstrating differentially expressed genes in primary hepatocytes treated with FA with or without pTreA40. Gene Ontology pathways significantly changed by TreA40 exposure are shown at *right*, *p* < 0.05. *G*, pTreA40 blocks CD53 expression in isolated hepatocytes untreated, treated with 25 mM fructose, or 500 μM BSA-conjugated FA. ∗, ∗∗, ∗∗∗, and ∗∗∗∗ *p* < 0.05, *p* < 0.01, *p* < 0.001, *p* < 0.0001 by one-way ANOVA with Dunnett’s post hoc testing. AMPK, AMP-activated protein kinase; FGF21, fibroblast growth factor 21; NAMPT, nicotinamide phosphoribosyltransferase; PGC1α, peroxisome proliferator antigen receptor gamma coactivator-1-alpha; TreA, 6-O-acryloyl-trehalose; UCP1, uncoupling protein 1.

We therefore sought to define compounds that block CD53 expression in hepatocytes. This effort was in response to our observations that (i) germline CD53 deletion improves hepatic and extrahepatic metabolic and inflammatory status in mice and (ii) CD53 inhibition in hepatocyte cell-autonomously improves hepatic lipid accumulation and inflammatory signaling. Here, we demonstrated efficacy of GLUT8 blockade and trehalose treatment as effective means to protect against FA + LPS-induced hepatocyte CD53 expression. However, a potential limitation for GLUT8 inhibitors or trehalose as novel human therapies is that selective GLUT8 inhibitors are not yet defined, and trehalose is labile to intestinal brush border trehalases that hydrolyze trehalose into component glucose monomers (40, 41, 52). We previously showed that lactotrehalose is a trehalose analog that differs in its carbon four hydroxyl group orientation, and this confers trehalase resistance and greater potency to induce hepatocyte fasting–like responses (Fig. 7*A*, and (40, 41, 51)).

We therefore tested efficacy of trehalose and lactotrehalose in comparison to a set of trehalose polymers—pTreA20 and pTreA40 (Fig. 7*A*). We explored the therapeutic utility of trehalose polymers based on three main observations. First, free trehalose induces autophagic flux, blocks GLUTs, and improves hepatic steatosis. Second, trehalase-resistant trehalose analogs augment bioavailability and potency over trehalase-labile trehaloses (40, 52). Third, trehalose polymers, pTreA20 and pTreA40, resist degradation by trehalases (66). On these bases, we tested the hypothesis that trehalase-resistant polymers induce fasting-like hepatocellular signals.

pTreA20 and pTreA40 polymers consist of 20 or 40 units of free trehalose, respectively (Fig. 7*A*). Both were synthesized using acrylate monomers, and their polymer backbones are identical. The primary difference between each of these polymers is the density of trehalose units pending from polymer backbone. pTreA40 is a trehalose-rich polymer containing 85.1% w/w of trehalose. pTreA20 contains 61.5% w/w of trehalose (Table 1), and half of trehalose units were exchanged with inert 2-hydroxyethyl acrylate (HEA) monomer. This effectively lowers the density trehalose units in pTreA20 *versus* pTreA40 (Fig. 7*A*). We delivered these polymers while controlling for total carbohydrate content (at 100 mM), such that differential responses to pTreA20 and pTreA40 are generally attributable to the trehalose density, rather than absolute trehalose content. pTreA20 and pTreA40 are waters soluble and resist brush border trehalases because they cannot be enzymatically liberated from their carbon scaffold as we previously demonstrated (66). Their theoretical molar mass and relative trehalose content are shown in Table 1, and corresponding [^1^H]-NMR spectra used to derive these data are demonstrated in Fig. S4.

**Table 1:** Characterization of trehalose polymers

Polymer^a^	Conversion (%)	M_n__theoretical_^a^ (kDa)	Trehalose content (% w/w)
pTreA_40_	98	16,098	85.1
pTreA_20_	98	11,107	61.5

a Calculated based on ^1^H NMR spectra.

Free trehalose blocks carbohydrate uptake into hepatocytes as a nonselective GLUT inhibitor (31, 34, 35, 40, 41). We therefore examined first whether trehalose polymer also inhibits hepatocyte carbohydrate transport. We pretreated WT primary hepatocytes with pTreA20 (30 min) and then quantified radiolabeled [^3^H]-2-deoxy-d-glucose ([^3^H]-2DG) and [^3^H]-fructose uptake (Fig. S5). Free trehalose blocked [^3^H]-2DG uptake as we reported previously (35). A nonspecific carbohydrate polymer control, sucrose polymer (pSucA20), did not inhibit [^3^H]-2DG transport. In contrast, trehalose polymer, pTreA20, blocked uptake of both radiolabeled substrates, [^3^H]-2DG and [^3^H]-fructose (Fig. S5). We next asked if blocking carbohydrate uptake in hepatocytes conferred enhanced fasting-like signal transduction in hepatocytes. Basal fed-state treatment in isolated hepatocytes with pTreA20 and pTreA40 induced FGF21, PGC1α, and UCP1, with pTreA40 activating these to a significantly greater magnitude than trehalose (Fig. 7*B*). In addition to UCP1, pTreA20 induced UCP2, and pTreA40 induced both UCP2 and UCP5 when compared with untreated and free trehalose-treated cultures (Fig. S6). When challenged with hyperalimentation, trehalose, LactoTre, and pTreA40 overcame the presence of excess (25 mM) fructose (Fig. 7*C*) and (500 μM) BSA-conjugated FAs (Fig. 7*D*) to increase fasting target gene expression. In addition, pTreA40 activated accumulation of the autophagic flux marker protein, LC3B-II, and phosphorylation of the AMPK (T172) (Fig. 7*E*), even in the presence of excess conjugated FA. Transcriptomic analysis of FA-exposed hepatocytes in the presence or the absence of pTreA40 revealed activated unsaturated FA metabolism, lipid metabolic processes, and downregulation of innate immune responses, cell stress responses, and viral/interferon-β responses (Fig. 7*F*). In light of these metabolic, signaling, and transcriptomic changes in pTreA40-treated hepatocytes, we demonstrated that pTreA40 inhibits hepatocyte CD53 expression in the basal, fructose-fed, and FA-exposed states. Together, we identify pTreA40 as a stabilized trehalose-derivative polymer that induces enhanced fasting like signaling and suppresses hepatocyte CD53 with potency that exceeds that of free trehalose.

## Discussion

Tetraspanins are ubiquitous membrane-spanning proteins that traditionally serve as inflammatory mediators. CD53 is an incompletely characterized tetraspanin previously implicated in B-cell development, lymphocyte trafficking, and inflammatory disease. However, the broad expression profile for CD53 and the identification of CD53 in genome-wide studies in fatty liver and obesity indicated CD53 functions that extend beyond inflammatory cell activation.

Here, we provide unexpected evidence that CD53 functions at the interface of metabolism and inflammation. We assert this based on multiple lines of evidence uncovered herein. First, both inflammatory and metabolic perturbations cell-autonomously regulate CD53 expression in hepatocytes. In particular, CD53 expression correlated with overall overnutrition or fasting-like status in the liver in mice or in isolated cultured hepatocytes. Second, CD53 ablation blocked diet-induced liver lipid and glucose homeostatic perturbations in NASH-inducing contexts but not in the setting of high-fat (western style) dietary feeding alone (although it is noted that CD53 KO mice were protected from WD-induced hypertriglyceridemia). Third, we know that this protection from metabolic stress is independent of impaired B-cell and T-cell function and independent of generalized immunosuppression *per se* because isolated CD53-deficient hepatocytes were protected from FA- and LPS-induced TG accumulation. Fourth, concomitant with these metabolic protections, CD53 ablation blocked liver and adipose inflammatory transcriptomic responses in mice under diet-induced duress. Finally, independent and untargeted proteomic data corroborate physiological, transcriptomic, and biochemical data to indicate that hepatocyte CD53 interacts with signaling intermediaries in both metabolic and inflammatory cascades upon FA + LPS stimulation. We interpret these data to indicate that CD53 function integrates metabolic and inflammatory status in hepatocytes. Yet, we acknowledge that further defining hepatocyte-specific functions in our dietary NASH model *in vivo* will require tissue-specific genetic interrogations *in vivo*. This will clarify whether deleting CD53 in adipose compartment, lymphocyte compartment, or both compartments will reproduce the protective effects of generalized CD53 deletion on peripheral adiposity in high-fat diet–fed contexts.

Understanding these data in light of prior literature on CD53 function raises interesting points for further investigation, particularly with regard to fundamental NASH pathophysiology. For example, CD53-deficient mice have low total B-cell number and have defective B-cell and T-cell homing to lymph nodes (6, 7, 8). Given that CD53 KO mice exhibited similar overall NASH scoring in our dietary NASH model, one key pathophysiological insight here is that full lymphocyte development, homing, migration, and function is dispensable for histological NASH progression. This aligns in part with recent data, which suggest that gut-derived metabolites (*e.g.*, LPS) activate intrahepatic B lymphocytes to induce NASH (60, 67, 68, 69). Prior data suggest that a primary function of CD53 is in lymphocyte homing, whereas it is the resident, or intrahepatic, B-cells that are considered primarily to mediate NASH pathology (60, 67, 68, 69). Our data suggest either (i) CD53^+^ and intrahepatic B-cell subsets represent distinct subpopulations in NASH pathophysiology or (ii) there is functional redundancy, in which other cell types drive hepatic inflammation in NASH in the absence of a full lymphocyte complement. Nevertheless, data here extend prior literature to suggest that hepatocyte CD53, not lymphoid CD53, mediates both metabolic and inflammatory signaling independent of lymphoid cell CD53 function. Future interrogation will better distinguish the relative contributions in each compartment to the metabolic and inflammatory protections afforded in the CD53 KO mouse model.

In addition to new biology elucidated here, we defined therapeutic options to block CD53 expression. Because CD53 expression demarcates nutrient status, we showed that each of genetic GLUT blockade, trehalose treatment, and lactotrehalose blocked CD53 expression. This CD53 inhibition directly correlates with inhibited hexose uptake and diet-induced hepatic steatosis *in vivo*. Given that both genetic GLUT8 blockade and generalized nonselective pharmacologic GLUT inhibition similarly inhibited CD53 expression, we conclude that specific GLUT8 blockade is not required to inhibit CD53. Yet, whether CD53 mechanistically participates in the adaptive metabolic effects, which we previously demonstrated in GLUT8-deficient mice (*e.g.*, protection from NAFLD and insulin resistance (32, 33, 38, 44, 53, 70, 71, 72)), remains to be fully determined. Nevertheless, we introduced a novel scaffolded trehalose polymer, which is a unique composition of matter, and water-soluble compound that induces hepatocyte fasting–like signaling responses, and inhibits CD53 expression in unperturbed, fructose-, and FA-treated hepatocytes to an equal or greater extent than trehalose.

Together, we show that CD53 mediates metabolic and inflammatory functions in hepatocytes and adipose tissue. Not only is CD53 blockade protective against diet-induced peripheral and hepatic lipid accumulation, and against impaired glucose homeostasis, but also it mediates TNFα and LPS signaling in hepatocytes independent of the immune compartment. Finally, we introduce fasting signal-inducing trehalose polymers to modulate CD53 expression. The data provide insights into lymphocyte function during NASH, identify a novel target, and offer a potential new treatment to combat inflammatory disorders of overnutrition.

## Experimental procedures

### Animal studies

#### Mice, diets, and treatments

All animal protocols were approved by the Washington University School of Medicine Animal Studies Committee. Male C57B/6J mice were purchased directly from the Jackson Laboratory. Targeted CD53 KO mice (8) and liver-specific GLUT8 KO mice (40) were generated as previously described.

All strains of genetically altered mice were maintained on a C57BL/6J background. Control mice were littermates and thus matched by genetic background, age, and sex. All animals were housed at the Washington University Medical School in St Louis in a 12-h alternating light–dark, temperature-controlled, specific pathogen-free barrier facility prior to and throughout experimentation.

All animals received humane care, and procedures were performed in accordance with the approved guidelines by the Animal Studies Committee at Washington University School of Medicine. All animal studies were performed in accordance with the criteria and ethical regulations outlined by the Institutional Animal Care and Use Committee.

Five-week-old mice were fed ad libitum: a normal chow diet or a WD (TD.88137: 42% kcal fat; Envigo Teklad Diets) for 16 weeks with or without low-dose weekly intraperitoneal carbon tetrachloride (0.32 μg/g BW) administration and fructose/glucose (23.1 and 18.9 g/l) water as previously described (64). All animals received nonsupplemented drinking water.

### Primary murine hepatocytes

Primary murine hepatocytes obtained from WT mice were isolated (24, 25, 31) and cultured and maintained in regular Dulbecco's modified Eagle's growth medium (Sigma; catalog no.: D5796) containing 10% fetal bovine serum. Briefly, we plated 1 × 10^6^ cells per well in 6-well plates, which yielded 70 to 80% confluent plates for experimentation. About 12 h after plating, cells were treated with fructose or 500 μM BSA-conjugated FA in the presence or the absence of trehalose, LT, or polymer for 24 h prior to assay. All cultures were lysed in Trizol and subjected to downstream analysis. Trehalose polymer and lactotrehalose were generated as described (40, 66). Recombinant murine TNFα was obtained from Sigma–Aldrich (catalog no.: T7539) and used at 100 ng/ml *in vitro*.

#### Synthesis of trehalose polymers

pTreA40 and pTreA20 were synthesized by reversible addition-fragmentation chain transfer polymerization according to the procedure described previously (32). Reversible addition-fragmentation chain transfer polymerization was carried out in dimethyl sulfoxide (DMSO using 2-hydroxyethyl carbonotrithioate as chain transfer agent (CTA) and 2,2′-azobis(2-methylpropionitrile) (AIBN) as thermal initiator. For the synthesis of pTre40, 6-O-acryloyl-trehalose (TreA) (8.0 × 10^−4^ mol) was weighed in 4 ml glass vials, to which 200 μl of DMSO, 2.0 × 10^−5^ mol of CTA (400 μl of a 50 mM stock solution in DMSO), and 4.0 × 10^−6^ mol of AIBN (200 μl of a 20 mM stock solution in DMSO) were added, such that [TreA]:[CTA]:[AIBN] = 40:1:0.2. The vials were each sealed with a silicone septum, and the solutions were thoroughly deoxygenated with argon for 1 h. Each vial was then transferred to an oil bath preheated to 70 °C and stirred (400 rpm) under these conditions for 16 h. The polymerization was stopped by cooling the vials in an ice bath and exposing the polymerization solutions to air. The monomer conversion was calculated based on the ^1^H-NMR spectrum of the crude products in D_2_O. The polymer was purified by dialysis (Spectra/Por 6; molecular weight cutoff: 1 kDa, 18 mm) against deionized water (∼2.5 l, changed three times) for 24 h and then freeze-dried to afford pTreA_40_ as pale yellow powder. For the synthesis of pTre20, polymerization was carried out in the presence of HEA, and molar ratio of reagents was as follows: [TreA]:[HEA]:[CTA]:[AIBN] = 20:20:1:0.2.

#### Intraperitoneal glucose tolerance test

Intraperitoneal glucose tolerance tests were carried out on mice fasted for 6 h on aspen bedding. Basal blood glucose concentrations were determined for each mouse prior to glucose administration using a hand-held glucose meter (Arkray USA, Inc). Each mouse then received 2 g per kg BW of glucose, except for *db/db* mice, which received 1 g per kg BW of glucose through intraperitoneal injection, and blood glucose concentrations were subsequently measured at 30, 60, 90, and 120 min post glucose administration.

#### Intraperitoneal insulin tolerance test

Intraperitoneal insulin tolerance tests were carried out on mice fasted for 4 h on aspen bedding. Basal blood glucose concentrations were determined for each mouse prior to insulin administration using a hand-held glucose meter (Arkray USA, Inc). Each mouse then received 0.75 IU per kg BW of insulin (Lilly USA, LLC) through intraperitoneal injection, and blood glucose concentrations were subsequently measured at 30, 60, 90, and 120 min post insulin administration.

#### Clinical chemistry measurements and hepatic lipid analyses

For all other serum analyses, submandibular blood collection was performed immediately prior to sacrifice, and serum was separated. Insulin ELISA (Millipore; catalog no.: EZRMI-13K), TGs (Thermo Fisher Scientific; catalog no.: TR22421), cholesterol (Thermo Fisher Scientific; catalog no.: TR13421), and free FA (Wako Diagnostics; catalog nos.: 999-34691, 995-34791, 991-34891, and 993-35191) quantification were performed using commercially available reagents according to the manufacturer’s directions. Albumin levels were quantified using an AMS LIASYS Chemistry Analyzer.

#### Measurement of liver TGs

Liver-specific lipids were extracted and analyzed from snap-frozen liver tissue samples. About ∼50 mg hepatic tissue samples were homogenized in 2:1 chloroform:methanol. In total, 0.25 to 0.5% of each extract was evaporated overnight prior to biochemical quantification of TGs, cholesterol, and free FAs using reagents described previously, precisely according to the manufacturer’s directions.

#### Body composition analysis

Body composition analysis was carried out in unanesthetized mice using an EchoMRI 3-1 device (Echo Medical Systems) *via* the Washington University Diabetic Mouse Models Phenotyping Core Facility.

#### Indirect calorimetry and food intake measurement

All measurements were performed in a PhenoMaster System (TSE Systems) *via* the Washington University Diabetic Mouse Models Phenotyping Core Facility, which allowed metabolic performance measurement and activity monitoring by an infrared light  = beam frame. Mice were placed at room temperature (22–24 °C) in separate chambers of the PhenoMaster open-circuit calorimetry. Mice were allowed to acclimatize in the chambers for 4 h. Food and water were provided ad libitum in the appropriate devices. Respiratory exchange ratio was measured for at least 24 h for a minimum of one light cycle (6:01 AM to 6:00 PM) and one dark cycle (6:01 PM to 6:00 AM). Presented data are average values obtained in these recordings.

#### Quantitative real-time RT–PCR

Total RNA was prepared by homogenizing snap-frozen livers or cultured hepatocytes in Trizol reagent (Invitrogen; catalog no.: 15596026) according to the manufacturer’s protocol. Complementary DNA (cDNA) was prepared using Qiagen Quantitect reverse transcriptase kit (Qiagen; catalog no.: 205310). Real-time quantitative PCR was performed with Step-One Plus Real-Time PCR System (Applied Biosystems) using SYBR Green Master Mix Reagent (Applied Biosystems) and specific primer pairs. Relative gene expression was calculated by a comparative method using values normalized to the expression of an internal control gene.

#### Immunoblotting

Tissues were homogenized in radioimmunoprecipitation assay lysis buffer (50 mM Tris, 1% NP-40, 0.1% SDS, 0.5% sodium deoxycholate, 150 mM NaCl, pH 8.0) supplemented with protease and phosphatase inhibitors (Thermo Scientific). After homogenization, lysate was centrifuged at 18,000*g* for 15 min at 4 °C, and the supernatant was recovered. Protein concentration was determined by BCA Assay Kit (Thermo Scientific) and was adjusted to 2 mg/ml. Samples for Western blotting were prepared by adding Laemmli buffer at a ratio of 1:1 and heating at 95 °C for 5 min. The prepared samples were subjected to 10% or 13% SDS-PAGE, followed by electrical transfer onto a nitrocellulose membrane using the Trans-Blot Turbo system (Bio-Rad). After blocking the membrane with 5% milk in Tris-buffered saline with Tween-20, the membrane was incubated in primary antibody at 4 °C overnight. The blot was developed after secondary antibody incubation using Pierce ECL Western Blotting Substrate (Thermo Scientific). Blots were developed according to the manufacturer’s instructions. Protein expression levels were quantified with ImageJ Lab software (open-source software originated by the National Institutes of Health) and normalized to the levels of β-actin.

#### Histological analysis

Formalin-fixed paraffin-embedded liver sections were stained by H&E *via* the Washington University Digestive Diseases Research Core Center. Optimal cutting temperature–embedded frozen liver sections were stained by Oil Red O according to standard protocols flowered by microscopic examination. Three liver sections were examined and evaluated for each animal. For Oil Red O staining, ice-cold methanol-fixed frozen sections from mice were stained according to the described protocols (24, 25, 46).

### Immunofluorescent staining

Confocal microscopy was performed as reported (33) with some modifications. Paraffin sections were permeabilized and blocked in PBS containing 5% BSA, 0.2% gelatin 60′ at room temperature in a humid box. Rat antimouse CD53 antibody (1:100 dilution) was incubated with each section in 1% BSA, 0.2% gelatin overnight at RT. Primary antisera were rinsed with PBS/Triton/0.2% gelatin solution prior to staining using donkey antirat ALEXA Fluor 594 at 1:1000 dilution in PBS and 1% BSA with 4′,6-diamidino-2-phenylindole (1:1000 dilution) at room temperature prior to imaging.

#### Antibodies

Antibodies against AMPK (phospho-T172; Cell Signaling; catalog no.: 2535), LC3B (Novus Biologicals; catalog no.: NB100-2220), and GAPDH (Cell Signaling; catalog no.: 5174). The dilution ratio for all primary antibodies was 1:1000. The secondary antibodies used in this study were peroxidase-conjugated anti-rabbit immunoglobulin G (Cell Signaling; catalog no.: 7074S), which were used at a 1:5000 dilution.

#### RNA-Seq

RNA-Seq was performed by the Washington University Genome Technology Access Center as previously described (31, 46). Library preparation was performed with 10 μg of total RNA with a Bioanalyzer RIN score greater than 8.0. Ribosomal RNA was removed by poly-A selection using Oligo-dT beads (mRNA Direct kit; Life Technologies). mRNA was then fragmented in buffer containing 40 mM Tris acetate (pH 8.2), 100 mM potassium acetate, and 30 mM magnesium acetate and heating to 94° for 150 s. mRNA was reverse transcribed to yield cDNA using SuperScript III RT enzyme (Life Technologies, per manufacturer’s instructions) and random hexamers. A second strand reaction was performed to yield ds-cDNA. cDNA was blunt ended, had an A base added to the 3′ ends, and then had Illumina sequencing adapters ligated to the ends. Ligated fragments were then amplified for 12 cycles using primers incorporating unique index tags. Fragments were sequenced on an Illumina HiSeq-3000 using single reads extending 50 bases.

RNA-Seq reads were aligned to the Ensembl release 76 top-level assembly with STAR, version 2.0.4b (open source code originated by Dr Alex Dobin, Cold Spring Harbor Laboratory). Gene counts were derived from the number of uniquely aligned unambiguous reads by Subread:featureCount, version 1.4.5. Transcript counts were produced by Sailfish, version 0.6.3 (open-source software originated by Dr Rob Patro, Carnegie Mellon University). Sequencing performance was assessed for total number of aligned reads, total number of uniquely aligned reads, genes and transcripts detected, ribosomal fraction known junction saturation, and read distribution over known gene models with RSeQC, version 2.3 (open-source software originated by Dr Liguo Wang, Baylor College of Medicine).

To enhance the biological interpretation of the large set of transcripts, grouping of genes/transcripts based on functional similarity was achieved using the R/Bioconductor packages GAGE and Pathview (open source software originated by Dr Steven Salzberg, Johns Hopkins University and Weijun Luo, University of North Carolina, Charlotte). GAGE and Pathview were also used to generate pathway maps on known signaling and metabolism pathways curated by Kyoto Encyclopedia of Genes and Genomes.

#### Proteomic analysis

GFP-tagged CD53 was immunoprecipitated. Peptides were prepared after release of proteins from antibody beads. The beads were washed with 1 ml of 50 mM cold PBS (pH 7.4) followed by elution with 40 μl of SDS buffer (4% w/v, 100 mM Tris–HCl, pH 8.0). Protein disulfide bonds were reduced using 100 mM DTT with heating to 95 °C for 10 min. Peptides were prepared as previously described using a modification (PMID: 28188519) of the filter-aided sample preparation method (PMID: 19377485). Samples were mixed with 600 μl of 100 mM Tris–HCl buffer, pH 8.5 containing 8 M urea (UA buffer). They were transferred to the top chamber of a 30,000 molecular weight cutoff filtration unit (Millipore; part MRCF0R030) and processed to peptides as previously described (PMID: 28188519). The peptides were dried in a Speedvac concentrator (Savant DNA 120 Speedvac Concentrator; Thermo Scientific) for 15 min. Dried peptides were dissolved in 1% (v/v) TFA and desalted using Stage tips (PMID). The peptides were eluted with 60 μl of 60% (v/v) MeCN in 0.1% (v/v) TFA and dried in a Speed-Vac (Thermo Scientific; model no.: Savant DNA 120 concentrator). They were then dissolved in 20 μl of 1% (v/v) MeCN in water. An aliquot (10%) was removed for quantification using the Pierce Quantitative Fluorometric Peptide Assay kit (Thermo Scientific; catalog no.: 23290).

Peptides were analyzed using trapped ion mobility time-of-flight mass spectrometry (PMID: 30385480). Peptides were separated using a *nano-ELUTE* chromatograph (Bruker Daltonics) interfaced to a timsTOF Pro mass spectrometer (Bruker Daltonics) with a modified nano-electrospray source (CaptiveSpray; Bruker Daltonics). The mass spectrometer was operated in parallel accumulation–serial fragmentation mode (PMID: 30385480). The samples in 2 μl of 1% (v/v) FA were injected onto a 75 μm i.d. × 25 cm Aurora Series column with a CSI emitter (IonOpticks). The column temperature was set to 50 °C. The column was equilibrated using constant pressure (800 bar) with eight column volumes of solvent A (0.1% v/v FA). Sample loading was performed at constant pressure (800 bar) at a volume of one sample pick-up volume plus 2 μl. The peptides were eluted using one column separation mode with a flow rate of 300 nl/min and using solvents A (0.1% v/v FA) and B (0.1% v/v FA/MeCN): solvent A containing 2% B increased to 17% B over 60 min, to 25% B over 30 min, to 37% B over 10 min, to 80% B over 10 min and constant 80% B for 10 min. The MS1 and MS2 spectra were recorded from *m/z* 100 to 1700.

The collision energy was ramped stepwise as a function of increasing ion mobility: 52 eV for 0 to 19% of the ramp time; 47 eV from 19 to 38%; 42 eV from 38 to 57%; 37 eV from 57 to 76%; and 32 eV for the remainder. The TIMS elution voltage was calibrated linearly using the Agilent ESI-L Tuning Mix (*m/z* 622, 922, and 1222).

The MS2 spectra from peptides with +2, +3, and +4 charge states were analyzed using Mascot software (Perkins DN; PMID: 10612281) (version 2.5.1; Matrix Science). Mascot was set up to search against a custom and nonredundant UniProt (version: March 2021) database of mouse proteins (16,997 entries), assuming the digestion enzyme was trypsin with a maximum of four missed cleavages allowed. The searches were performed with a fragment ion mass tolerance of 50 ppm and a parent ion tolerance of 25 ppm. Carbamidomethylation of cysteine was specified in Mascot as a fixed modification. Deamidation of asparagine, deamidation of glutamine, pyroglutamate formation from N-terminal glutamine, acetylation of protein N terminus, and oxidation of methionine were specified as variable modifications. Peptides were filtered at 1% false discovery rate by searching against a reversed protein sequence database, and a minimum of two peptides were required for protein identification.

### Statistical analyses

Data were analyzed using GraphPad Prism, version 7.05 (Research Resource Identifier: SCR_015807). *p* < 0.05 was defined as statistically significant. Data shown are as mean ± SEM. Two-tailed homoscedastic *t* tests were used in pairwise comparisons. When more than one statistical comparison was performed, one-way ANOVA and two-way ANOVA were used for all analyses, as noted in the figure legends. All quantitative RT–PCR analyses were also analyzed using nonparametric assumptions. The results of each of these analyses are provided in Table S1.

For RNA-Seq analyses, we used Limma generalized linear models with contrast matrices and moderated *t*-statistics that allow for multiple testing and comparison corrections across all the genes and contrasts performed within the same statistical model. These are reported as the Benjamini–Hochberg adjusted *p* values. GO analyses used the R/Bioconductor GAGE package that performs a GO term level *t* test on the log_2_ fold changes on every gene within a term and tested in the transcriptomic analysis against the background of all other genes outside the term.

## Data and resource availability

There are no restrictions on data and material availability. Trehalose polymers were obtained under a material transfer agreement with Dr Wandzik. The data that support the findings of this study are available from the authors on reasonable request.

## Supporting information

This article contains supporting information.

## Conflict of interest

The authors declare that they have no conflicts of interest with the contents of this article.
