# Supplementary material for: The tetraspanin transmembrane protein CD53 mediates dyslipidemia and integrates inflammatory and metabolic signaling in hepatocytes
Source: J Biol Chem. 2022 Dec 27;299(2):102835. doi: 10.1016/j.jbc.2022.102835 (PMC9900517; doi:10.1016/j.jbc.2022.102835)
Supplement: Supplementary Figures [file mmc2.pdf]

Supp. Fig. 1

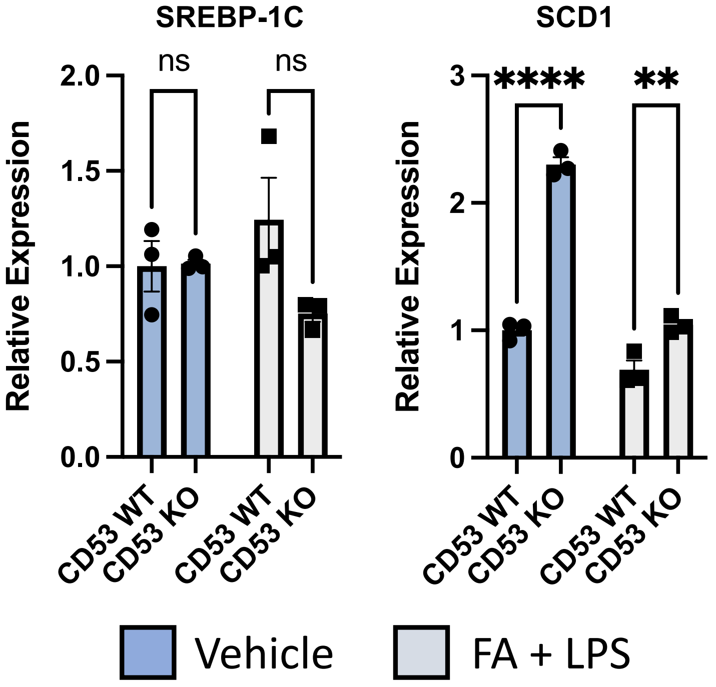

Supplemental Figure 1. SREBP-1C and SCD1 gene expression in isolated hepatocytes from CD53 WT and CD53 KO mice treated with or without FA + LPS. **, ****, $P < 0.01$, < 0.0001 versus bracketed control by two-way ANOVA with Sidak's post hoc testing.

Supp. Fig. 2

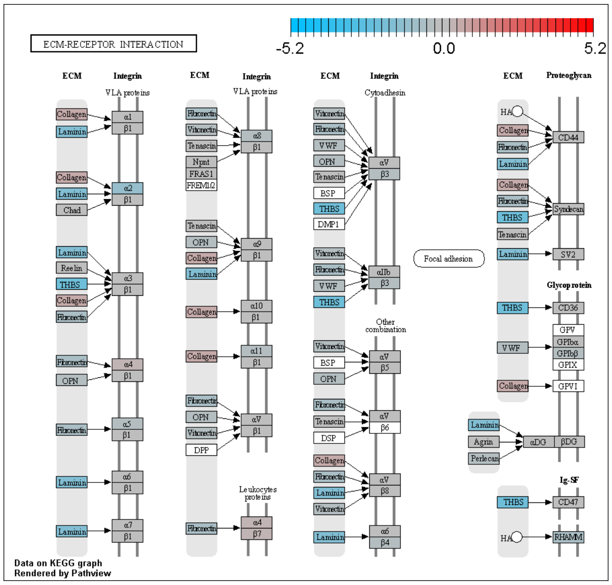

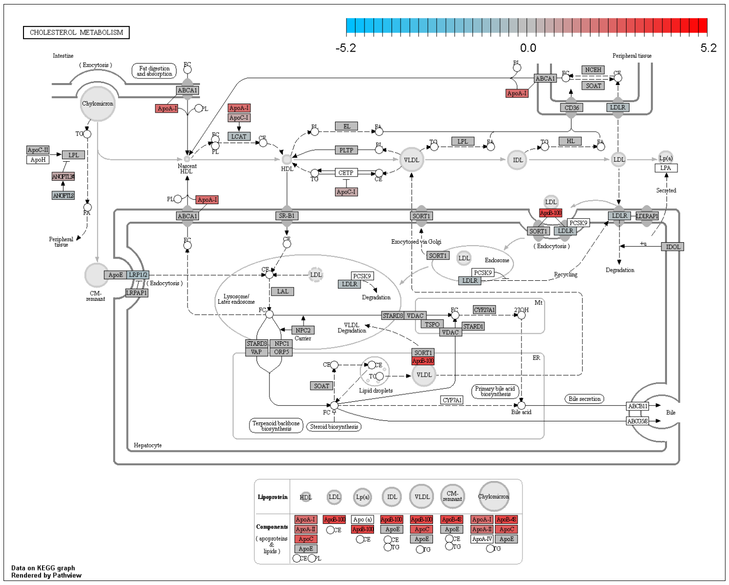

Supp. Fig 2. CD53 KO eWAT exhibits impaired ECM-receptor signaling and increased cholesterol metabolism. Left ECM-receptor interaction pathway heatmap diagram, and right, cholesterol metabolism diagram showing increased cholesterol metabolism in CD53 KO eWAT.

Supp. Fig. 3.

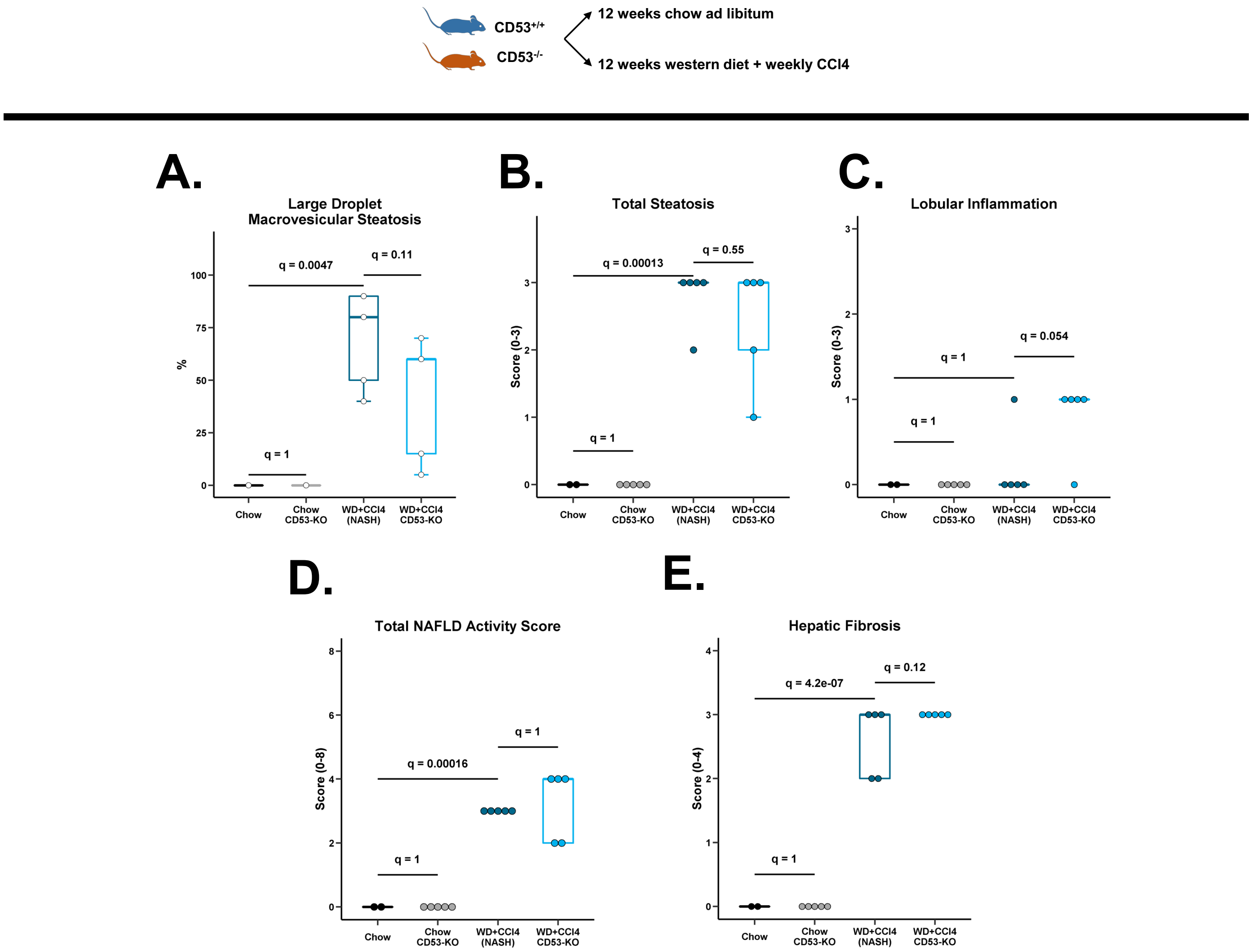

Supplemental Figure 3. Full-body CD53 KO protects from select metabolic pathologies. A-B. Steatosis scores in WT and CD53 KO livers treated with chow diet or western diet and weekly CCl4 injection (NASH-affected) (n = 4, 5, 5, 5, in order as pictured, and groups maintained throughout the figure). **C.** lobular inflammation, (**D.**), total NAFLD activity scores (**E.**), and fibrosis scores measured by blinded histological analysis.

Supp. Fig. 4.

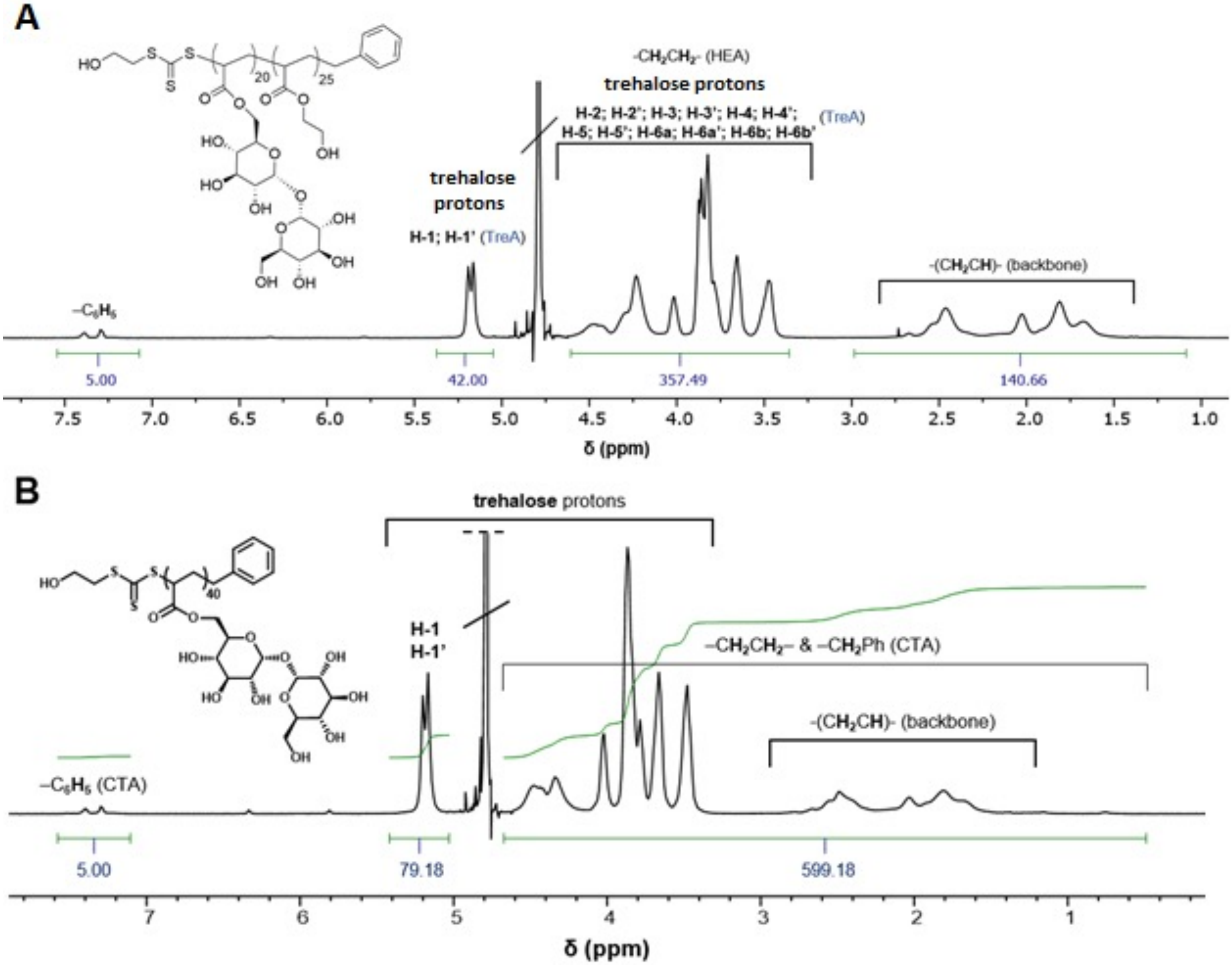

Supplemental Figure 4. ^{1}H NMR spectra of (A) pTreA20 and (B) pTreA40 (600 MHz, D_2O)

Supp. Fig. 5.

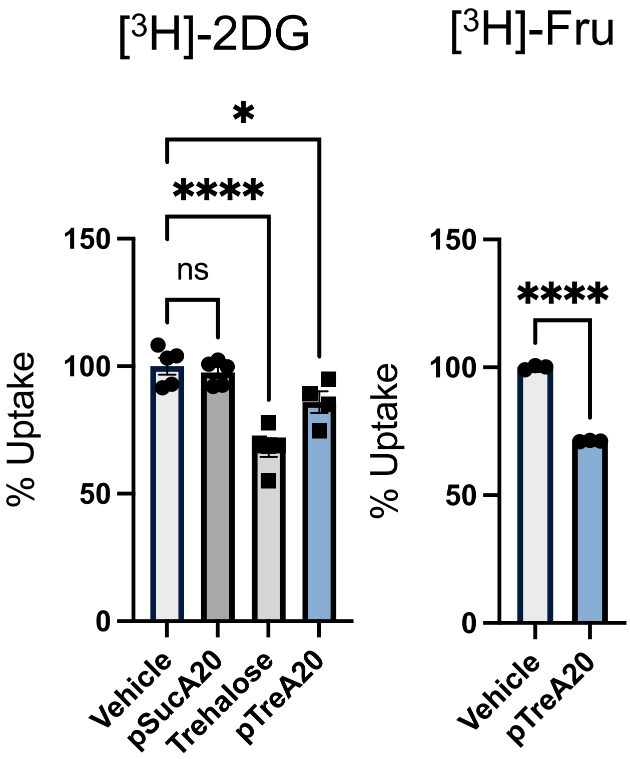

Supplemental Figure 5. ^{3}H-2DG and ^{3}H-fructose uptake in primary murine hepatocytes pre-incubated (30min) with vehicle or with 100mM polymeric sucrose (pSucA20), monomeric trehalose, or polymeric trehalose (pTreA20) prior to assay. Shown is uptake as % control uptake. *, **** P<0.05, < 0.0001 by one-way ANOVA with Dunnett's multiple comparison post hoc testing (left) and 2-tailed T-test (right) vs. bracketed controls.

Supp. Fig. 6.

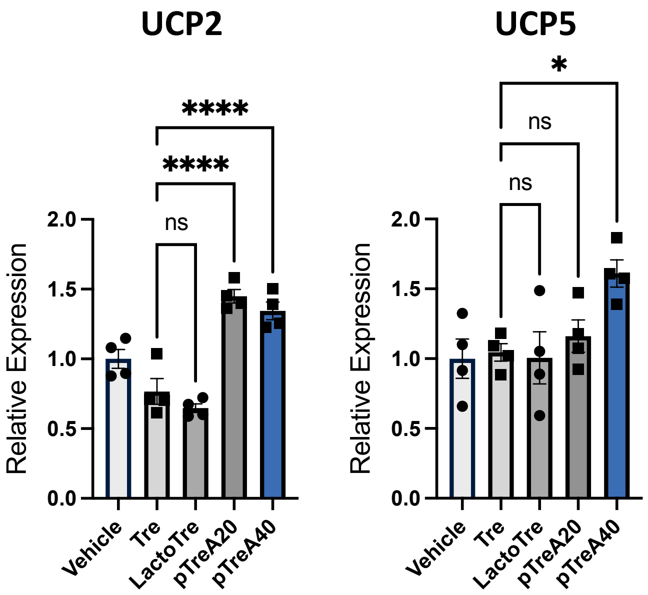

Supplemental Figure 6. Comparison of monomeric and polymeric trehalose effects on UCP2 and UCP5 expression in isolated hepatocytes. Shown are qRT-PCR analyses of total RNA from isolated primary murine hepatocyte cultures treated with vehicle, monomeric trehalose, lactotrehalose, or polymeric trehalose (24h, 100mM for each compound). *, ****, $P < 0.05$, < 0.0001 versus bracketed control by one-way ANOVA with Dunnett's multiple comparison post hoc testing.
