# Supplementary material for: The tetraspanin transmembrane protein CD53 mediates dyslipidemia and integrates inflammatory and metabolic signaling in hepatocytes
Source: J Biol Chem. 2022 Dec 27;299(2):102835. doi: 10.1016/j.jbc.2022.102835 (PMC9900517; doi:10.1016/j.jbc.2022.102835)
Supplement: Supplementary tables [file mmc3.pdf]

Figure	Panel	Group 1	Group 2	Group 3	Non-Parametric Test	Significance	Multiple Comparison Test	Group 1 vs 2	Group 2 vs 3
1	A	WT Chow	WT WD		Mann-Whitney	*			
	B	WT Chow	WT HTFC		Mann-Whitney	**			
	C	WT Chow	WT MCD		Mann-Whitney	***			
	D	Control	LPS	LPS + Trehalose	Kruskal-Wallis	****	Dunn's	**	*
	E - Female	Chow	NASH-D		Mann-Whitney	ns			
	E - Male	Chow	NASH-D		Mann-Whitney	p = 0.07			
	F - Veh	GLUT8 WT	GLUT8 WT + FA+LPS	GLUT8 LKO + FA+LPS	Kruskal-Wallis	**	Dunn's	ns	*
2	A - Liver	CD53 WT	CD53 KO		Mann-Whitney	*			
	A - Adipose	CD53 WT	CD53 KO		Mann-Whitney	*			
3	A - GPAT	WT - TNFa	CD53 KO - TNFa		Mann-Whitney	ns			
	A - LPK	WT - TNFa	CD53 KO - TNFa		Mann-Whitney	ns			
	A - SREBP-1C	WT - TNFa	CD53 KO - TNFa		Mann-Whitney	ns			
	A - ChREBP	WT - TNFa	CD53 KO - TNFa		Mann-Whitney	ns			
	A - ACC1	WT - TNFa	CD53 KO - TNFa		Mann-Whitney	ns			
	C - TNFa	WT Untreated	WT + TNFa	CD53 KO - TNFa	Kruskal-Wallis	**	Dunn's	*	ns
	C - CCL2	WT Untreated	WT + TNFa	CD53 KO - TNFa	Kruskal-Wallis	*	Dunn's	*	ns
	C - CXCL2	WT Untreated	WT + TNFa	CD53 KO - TNFa	Kruskal-Wallis	**	Dunn's	ns	*
	C - CLEC7A	WT Untreated	WT + TNFa	CD53 KO - TNFa	Kruskal-Wallis	**	Dunn's	ns	*
	C - NF-KB	WT Untreated	WT + TNFa	CD53 KO - TNFa	Kruskal-Wallis	**	Dunn's	ns	*
	C - IL-1B	WT Untreated	WT + TNFa	CD53 KO - TNFa	Kruskal-Wallis	*	Dunn's	*	ns
4	A - CD36	WT Untreated	WT - FA+LPS	CD53 KO - FA+LPS	Kruskal-Wallis	*	Dunn's	ns	ns
	A - LPK	WT Untreated	WT - FA+LPS	CD53 KO - FA+LPS	Kruskal-Wallis	*	Dunn's	*	ns
	A - ACC1	WT Untreated	WT - FA+LPS	CD53 KO - FA+LPS	Kruskal-Wallis	p = 0.07	Dunn's	ns	ns
	A - GPAT	WT Untreated	WT - FA+LPS	CD53 KO - FA+LPS	Kruskal-Wallis	*	Dunn's	ns	*
	A - ChREBP	WT Untreated	WT - FA+LPS	CD53 KO - FA+LPS	Kruskal-Wallis	*	Dunn's	ns	ns
	B - TNFa	WT Untreated	WT - FA+LPS	CD53 KO - FA+LPS	Kruskal-Wallis	*	Dunn's	*	ns
	B - CCL2	WT Untreated	WT - FA+LPS	CD53 KO - FA+LPS	Kruskal-Wallis	*	Dunn's	ns	*
	B - CXCL2	WT Untreated	WT - FA+LPS	CD53 KO - FA+LPS	Kruskal-Wallis	**	Dunn's	*	ns
	B - CXCL9	WT Untreated	WT - FA+LPS	CD53 KO - FA+LPS	Kruskal-Wallis	*	Dunn's	ns	ns
6	A - Chow CD53	CD53 WT	CD53 KO		Mann-Whitney	*			
	A - NASH-D CD53	CD53 WT	CD53 KO		Mann-Whitney	**			
	B	CD53 WT Chow	CD53 WT NASH-D	CD53 KO NASH-D	Mann-Whitney	*			
7	B - FGF21	Tre	pTreA20	pTreA40	Kruskal-Wallis	***	Dunn's	ns	**
	B - PGC1a	Tre	pTreA20	pTreA40	Kruskal-Wallis	**	Dunn's	ns	*
	B - UCP1	Tre	pTreA20	pTreA40	Kruskal-Wallis	***	Dunn's	ns	**
	C - FGF21	Fructose	pTreA40		Mann-Whitney	*			
	C - PGC1a	Fructose	pTreA40		Mann-Whitney	*			
	C - NAMPT	Fructose	pTreA40		Mann-Whitney	*			
	C - Arg2	Fructose	pTreA40		Mann-Whitney	*			
	C - UCP1	Fructose	pTreA40		Mann-Whitney	*			
	D - FGF21	FFA	pTreA40		Mann-Whitney	*			
	D - PGC1a	FFA	pTreA40		Mann-Whitney	*			
	D - NAMPT	FFA	pTreA40		Mann-Whitney	*			
	D - Arg2	FFA	pTreA40		Mann-Whitney	*			
	D - UCP1	FFA	pTreA40		Mann-Whitney	*			
	G - CD53 Basal	Vehicle	pTreA40		Mann-Whitney	*			
	G - CD53 Fructose	Vehicle	Fructose	Fructose + pTreA40	Kruskal-Wallis	**	Dunn's	ns	ns
	G - CD53 FA	BSA	FA	FA + pTreA40	Kruskal-Wallis	***	Dunn's	ns	**
Supp. 1	SREBP-1C	WT Untreated	WT - FA+LPS	CD53 KO - FA+LPS	Kruskal-Wallis	ns	Dunn's	ns	ns
	SCD1	WT Untreated	WT - FA+LPS	CD53 KO - FA+LPS	Kruskal-Wallis	p = 0.07	Dunn's	p = 0.07	ns
Supp. 6	UCP2	Vehicle	pTreA20	pTreA40	Kruskal-Wallis	**	Dunn's	*	ns
	UCP5	Vehicle	pTreA20	pTreA40	Kruskal-Wallis	*	Dunn's	ns	*

Supplemental Table 1. Non-parametric analyses of all gene expression data presented in Main and Supplemental Figures. *, **, ***, ****, P < 0.05, < 0.01, < 0.001, < 0.0001 versus indicated reference group by designated statistical test.
